# Informed choice and routinization of the second-trimester anomaly scan: a national cohort study in the Netherlands

**DOI:** 10.1186/s12884-023-05981-z

**Published:** 2023-09-26

**Authors:** Eline E.R. Lust, Kim Bronsgeest, Lidewij Henneman, Neeltje Crombag, Caterina M. Bilardo, Elsbeth H. van Vliet-Lachotzki, Robert-Jan H. Galjaard, Esther Sikkel, Monique C. Haak, Mireille N. Bekker

**Affiliations:** 1 Department of Obstetrics and Gynaecology, University Medical Center Utrecht, Utrecht, 3508 AB The Netherlands; 2 Department of Obstetrics and Gynaecology, Leiden University Medical Center, Leiden, The Netherlands; 3 Department of Human Genetics and Amsterdam Reproduction and Development Research Institute, Amsterdam UMC, location Vrije Universiteit Amsterdam, Amsterdam, The Netherlands; 4 Department of Obstetrics and Gynaecology, Amsterdam UMC, University of Amsterdam, Amsterdam, the Netherlands; 5 VSOP—Patient Alliance for Rare and Genetic Diseases, Soest, The Netherlands; 6 Department of Clinical Genetics, Erasmus University Medical Center, Rotterdam, The Netherlands; 7 Department of Obstetrics and Gynaecology, Radboud University Medical Center, Nijmegen, The Netherlands

**Keywords:** Informed choice, Second-trimester anomaly scan, Mid-trimester scan, Decision making, Reproductive autonomy

## Abstract

**Background:**

Since 2007 all pregnant women in the Netherlands are offered the second-trimester anomaly scan (SAS) in a nationwide prenatal screening program. This study aims to assess the level of informed choice of women opting for the SAS and to evaluate the presence of routinization 16 years after its implementation. It further explores decisional conflict and women’s decision making.

**Methods:**

This prospective national survey study consisted of an online questionnaire which was completed after prenatal counseling and before undergoing the SAS. Informed choice was measured by the adapted multidimensional measure of informed choice (MMIC) and was defined in case women were classified as value-consistent, if their decision for the SAS was deliberated and made with sufficient knowledge.

**Results:**

A total of 894/1167 (76.6%) women completed the questionnaire. Overall, 54.8% made an informed choice, 89.6% had good knowledge, 59.8% had deliberated their choice and 92.7% held a positive attitude towards the SAS. Women with low educational attainment (*p*=0.004) or respondents of non-Western descent (*p*=0.038) were less likely to make an informed choice. Decisional conflict was low, with a significantly lower decisional conflict score in women that made an informed choice (*p*<0.001). Most respondents (97.9%) did not perceive pressure to undergo the SAS.

**Conclusions:**

Our study showed a relatively low rate of informed choice for the SAS, due to absence of deliberation. Therefore, some routinization seem to be present in the Netherlands. However, most women had sufficient knowledge, did not perceive pressure and experienced low decisional conflict.

**Supplementary Information:**

The online version contains supplementary material available at 10.1186/s12884-023-05981-z.

## Introduction

The second-trimester anomaly scan (SAS) screens for fetal structural anomalies at a gestational age (GA) of 18–21 weeks [1, 2]. Since 2007, the SAS has been part of a nationwide program for prenatal screening in The Netherlands, which is organized and monitored by the government [3] . The SAS is offered for free to all pregnant women in the Netherlands. Pregnant women with a higher than background risk for fetal anomalies, are offered an advanced follow up fetal anomaly scan in a tertiary center for prenatal diagnosis [4]. The registered uptake for the SAS is 85.7% over 2021 [5]. This percentage is calculated by dividing all pregnancies with a SAS by the total number of pregnancies that are registered in the national registration system for prenatal screening. In the total number of pregnancies, also pregnancies with an indication for an advanced follow up fetal anomaly scan, as well as pregnancies that resulted in a miscarriage or intrauterine fetal demise, are included. Therefore, the uptake of the SAS is assumed to be higher than 85.7% [5].

Ultrasound in general has been described by women as an integral part of antenatal care and an opportunity not to be missed. It offers them a chance to ‘meet’ their unborn child [6]. In prenatal screening, the aim is providing reproductive autonomy for (future) parents [7, 8]. Therefore, decisions about prenatal screening should be based on objective, high-quality information and made in accordance with their own norms and values [7–14]. Informed choice is a major element in enhancing reproductive autonomy and is related to less decisional conflict and more satisfaction later in pregnancy [11, 12]. Informed choice is defined as a decision based on relevant knowledge, consistent with the decision-maker’s values and behaviorally implemented [14].

In the Netherlands, the level of informed choice in women deciding for fetal aneuploidy screening is high [12, 15–17]. However, the level of informed choice for the SAS has not been studied. Earlier studies on SAS reported on women’s knowledge and expectations regarding the scan and were published before 2008. These studies did not include all dimensions of informed choice [18–20]. Furthermore, as the uptake of the SAS is high in The Netherlands, the question arises whether routinization is present. Routinization refers to concerns that having a prenatal test might be self-evident to pregnant women or that their choices are uninformed and without proper consideration [21].

Therefore, the aim of this study is to assess the level of informed choice and decisional conflict of Dutch pregnant women opting for the SAS. In addition, it further explores women’s perceived pressure and reasons for choosing the SAS.

## Methods

This prospective national survey study was approved by the medical ethical review committee Leiden-Den Haag-Delft (METC LDD) (N20.151).

### Setting

In the Netherlands, the National Institute for Public Health and the Environment (RIVM) is responsible for the nationwide program for prenatal screening [22]. This program includes the non-invasive prenatal test (NIPT) and SAS. Furthermore, since September 2021, the first-trimester anomaly scan is offered in context of the IMITAS study. The combined test is not performed anymore [23, 24]. Prenatal counseling is performed by certified obstetric healthcare professionals. To become a certified prenatal counsellor, a mandatory course has to be successfully finished. For a prenatal counseling session, 30 min is scheduled. Next to the counseling session, women receive an information brochure about screening for physical anomalies in pregnancy [25, 26]. If the sonographer has seen an indication of an anomaly on the SAS, women are referred to a tertiary center for prenatal diagnosis for an advanced follow up fetal anomaly scan [3].

This study was performed between March and August 2021 in 96 primary care midwifery practices, three sonography practices and two tertiary hospitals, all were distributed equally across the Netherlands. Participants that opted for the SAS were asked to participate in this survey study by their prenatal counsellor after counselling. The included women received a questionnaire by email from the research group at a GA of 11 + 0 weeks. Informed consent was incorporated in the survey. The survey had to be completed by the respondents before receiving the SAS at a GA of 14 + 6 weeks. Women with a miscarriage during study participation were excluded. Furthermore, pregnant women with a high risk for fetal anomalies (e.g. teratogenic medicine-use, previous child with an anomaly, pregnant with monozygotic twins, first or second-degree relative with a congenital anomaly) were excluded, as they are offered an advanced follow up fetal anomaly scan in a tertiary center for prenatal diagnosis.

### Questionnaire

The questionnaire was developed by members of the research team including three maternal fetal medicine specialists, a midwife, a health scientist and a representative from a patient alliance. The survey consisted of validated questionnaires complemented with questions developed by the research group (Appendix A).

### Measures

#### Informed choice

To assess informed choice, the adapted multidimensional measure of informed choice (MMIC) [27, 28] was used. It comprises the dimensions knowledge, attitude, test uptake, and deliberation. An overview of the different dimensions of informed choice is demonstrated in Table 1. Knowledge about the SAS was measured by six statements. The knowledge items were developed by the multidisciplinary research team and were based on the national information brochure about screening for physical anomalies [3] and on the knowledge measure developed by Schoonen et al. [29]. There were three answer options: “true”, “false”, or “I do not know”. Good knowledge was defined as ≥ 4/6 correct statements. The answer option “I do not know” was considered as incorrect [12, 15]. Women’s attitude towards the SAS was measured by asking them to score four bipolar adjective pairs on a 5-point scale (negative–positive, difficult–easy, frightening–not-frightening, not reassuring–reassuring) [12, 17]. A sum score of > 14 was categorized as having a positive attitude, a sum score < 10 as having a negative attitude and scores from 10 to 14 were categorized as neutral [17]. Women with a neutral attitude were excluded from the analysis of value-consistency [12, 30]. Attitude and test uptake were combined in order to assess value-consistency. In this study we included women who opted for the SAS after prenatal counselling. As a result, respondents with a positive attitude were considered as value-consistent and respondents with a negative attitude were classified as value-inconsistent. Deliberation was measured using the deliberation scale. Deliberation is the process of evaluating the alternatives and weighing up pros and cons [28]. The deliberation scale consists of six items with a five-point Likert scale ranging from 1 (“strongly disagree”) to 5 (“strongly agree”) [28]. In order to dichotomize into a deliberated or not deliberated choice for the SAS, the mid-point (18 points) was used as the cut-off [28]. Informed choice was defined in case women were classified as value-consistent, if their decision for the SAS was deliberated and made with sufficient knowledge.

**Table 1:** Dimensions of informed choice

	Description	Items	Range	Reliability^a^	Cut-off
Knowledge	Knowledge regarding characteristics of the SAS	6 true-false statements, ‘do not know’ was considered as incorrect	0–6	-	≥ 4
Attitude	Attitude towards SAS	Four 5-point Likert scale items	4–20	0.59	> 14 = positive < 10 = negative
Uptake	Intention to accept or decline the SAS	-	-	-	-
Value consistency	Consistency between value (attitude) and behaviour (test uptake)	Calculated	-	-	-
Deliberation	Weighing up the pros and cons of the SAS, considering consequences	Six items with a five-point Likert scale	6–30	0.74	≥ 18
Informed choice	A choice made with good knowledge, deliberated and value-consistent	Calculated	-	-	-

^a^Reliability was assessed using Cronbach’s alpha

#### Decisional Conflict Scale (DCS)

Decisional conflict was assessed by using the validated Dutch DCS questionnaire [31, 32]. It consists of 16 statements, each using a five-point Likert scale ranging from 0 (“strongly agree”) to 4 (“strongly disagree”). The DCS contains five subscales: informed, values clarity, support, uncertainty, effective decision. The total score of an individual can be calculated by adding the scores of the 16 statements, ranging from 0 to 4, dividing the sum by 16 and then multiplying it by 25 [31]. The result is a score ranging from 0 (no decisional conflict) to 100 (extremely high decisional conflict). Meaningful differences are described for a score below 25.0 and a score exceeding 37.5. Scores below 25.0 indicate low decisional conflict. Scores exceeding 37.5 are associated with decisional delay or uncertainty about the decision, indicating high decisional conflict [31]. Cronbach’s alpha for the total DCS score was 0.95.

#### Decision making

Multiple choice questions in the questionnaire assessed if the respondents perceived pressure to have the SAS, whether they received advice by their gynecologist or midwife to do the SAS and what the most important reason was to have the SAS.

The following sociodemographic variables were included: maternal age, parity, educational level, ethnicity, religion, gestational age and method of conception.

### Statistical analysis

Statistical data analysis was performed using IBM SPSS statistics version 25. Descriptive statistics were used to describe participant characteristics and outcomes. Normally distributed variables were described using mean ± Standard Deviation (SD), while variables without normal distribution were described using median and Interquartile Range (IQR). Differences between groups were analyzed with the Mann-Whitney U test for numerical variables without normal distribution. A multiple logistic regression analysis was performed to determine which variables were associated with making an informed choice. Differences were considered significant if the *P*-value was < 0.05.

## Results

In total, 894/1167 (76.6%) pregnant women completed the questionnaire and were included for analysis. It was not reported how many eligible pregnant women declined receiving the survey.

Of the 273 exclusions, 232 women did not complete the survey before GA 15 weeks, 31 exclusions were due to an indication for an advanced follow up fetal anomaly scan (e.g. high-risk pregnancy for fetal anomaly or abnormal dating scan) and ten were excluded because of a non-viable pregnancy at the time of the survey.

An overview of the respondents’ characteristics is presented in Table 2.

**Table 2:** Characteristics of the study population

Characteristics	
Maternal age, years (mean ± SD)	31.5 ± 4.1
GA at time of completing questionnaire (median, IQR), missing 2	12 + 0 (9 days)
Parity, n (%)	
Primiparous	340 (38.0)
Multiparous	554 (62.0)
Education level, n(%)^a^, missing 1	
Low	25 (2.8)
Intermediate	241 (27.0)
High	627 (70.2)
Ethnicity^b^	
Dutch	756 (84.6)
Other western	68 (7.6)
Non-western	70 (7.8)
Religious affiliation, n (%), missing 12	
Not religious	633 (71.8)
Religious	249 (28.2)
Method of conception^c^, missing 1	
Natural	789 (88.4)
Assisted	104 (11.6)

^a^Education levels categorized as low (no education or highest attained educational level primary school, low level of secondary school, lower vocational training); intermediate (middle vocational training or high level of secondary school) or high (higher vocational training or university/doctorate) [15].

^b^Ethnicity categorized as Dutch: both parents were born in the Netherlands; other Western: one or both parents were born in Europe (excluding Turkey), North America, Oceania, Indonesia or Japan; non-Western: one or both parents were born in Africa, Latin-America, Asia (excluding Indonesia or Japan) or Turkey. Maternal country of birth was leading if both parents were born abroad [15].

^c^Assisted conception: in vitro fertilization or intra-cytoplasmic sperm injection (*n*=25), intrauterine insemination (*n*=20), at-home insemination (*n*=7), ovulation-induction (*n*=43), preimplantation genetic diagnosis (*n*=8), oocyte donation (*n*=1)

### Informed choice

 Overall, 54.8% (*n* = 455) of the pregnant women made an informed choice for having the SAS. Of the total study population, 89.6% (*n* = 801) had good knowledge, 59.8% (*n* = 535) had deliberated their choice, 92.7% (*n* = 829) had a positive attitude and were therefore classified as value-consistent (7.0% had a neutral attitude and were excluded from the informed choice analysis). An uninformed choice was made in 45.2% (*n* = 376), due to no deliberation (77.1%, *n* = 290), insufficient knowledge (10.1%, *n* = 38) or both insufficient knowledge and no deliberation (12.2%, *n* = 46). Multiple logistic regression indicated that the variables education level and ethnicity were significant predictors of informed choice, when correcting for religion, maternal age, parity, gestational age at time of completing the questionnaire and method of conception. Pregnant women with low educational attainment (odds ratio (OR): 0.22, 95% CI: 0.08–0.61, *p* = 0.004) were less likely to make an informed choice for the SAS compared to women with high educational attainment. Respondents of non-Western descent (OR: 0.56, 95% CI: 0.32–0.97, *p* = 0.038) were less likely to make an informed choice compared to women of Dutch descent (Table 3). Subanalysis showed that an uninformed choice was made in 78.3% (*n* = 18) of women with low education, due to no deliberation (55.6%, *n* = 10), insufficient knowledge (22.2%, *n* = 4) or both insufficient knowledge and no deliberation (22.2%, *n* = 4). An uninformed choice was made in 60.3% (*n* = 38) of respondents of non-Western ethnicity, due to no deliberation (55.3%, *n* = 21), insufficient knowledge (26.3%, *n* = 10), both insufficient knowledge and no deliberation (15.8%, *n* = 6) or value-inconsistency (2.6%, *n* = 1).

**Table 3:** Multiple logistic regression: factors associated with making an informed choice (*n*=816)^a^

Variable	Odds ratio	(95%-CI)	*P*
Maternal age	0.99	(0.95–1.03)	0.578
GA at time of completing questionnaire	1.00	(1.00–1.00)	0.324
Parity			
Primiparous	0.76	(0.56–1.03)	0.075
Multiparous^b^			
Education level			
Low	0.22	(0.08–0.61)	0.004
Intermediate	0.78	(0.56–1.09)	0.145
High^b^			
Ethnicity			
Dutch^b^			
Other western	0.97	(0.58–1.63)	0.91
Non-western	0.56	(0.32–0.97)	0.038
Religious affiliation			
Not religious^b^			
Religious	1.06	(0.77–1.47)	0.728
Method of conception			
Natural^b^			
Assisted	0.95	(0.62–1.47)	0.829

Abbreviations: *CI* – Confidence Interval, *GA* – Gestational Age

^a^63 women were excluded from the informed choice analysis, because of a neutral attitude. In addition, 15 women were excluded in the multiple logistic regression analysis due to missing values on one of the variables

^b^Reference category

### Decisional Conflict Scale (DCS)

Table 4 shows the scores of the DCS. The median total score was 17.2 points (IQR 20.3), indicating low decisional conflict after choosing for the SAS. Respondents that made an informed choice had a significantly lower (*p* < 0.001) total DCS score (12.5; IQR 22.0) compared to respondents who made an uninformed choice (18.8; IQR 19.0). In addition, the median scores on the subscales “informed”, “values clarity” and “support” were significantly lower (*p* < 0.001) in the informed choice group compared to the uninformed choice group.

**Table 4:** Decisional Conflict in total study population and in relation to informed choice

		All (*n* = 894) Median (IQR)	Informed choice (*n* = 455) Median (IQR)	Uninformed choice (*n* = 376) Median (IQR)	*P*
Subscores	Informed	25.0 (25.0)	16.7 (25.0)	25.0 (25.0)	< 0.001
	Values Clarity	25.0 (33.0)	16.7 (25.0)	25.0 (25.0)	< 0.001
	Support	16.7 (25.0)	8.3 (25.0)	16.7 (25.0)	< 0.001
	Uncertainty	8.3 (25.0)	8.3 (25.0)	8.3 (25.0)	0.021
	Effective decision	12.5 (25.0)	12.5 (25.0)	12.5 (25.0)	0.002
	*Total score*	*17.2 (20.0)*	*12.5 (22.0)*	*18.8 (19.0)*	*< 0.001*

*IQR* - interquartile range

The lower the scores, the more informed/clearer/supported/certain/effective decision making the pregnant woman feels/experiences

Cronbach’s alpha total DCS score: 0.95

### Decision making

The majority of the respondents (97.9%, *n*=875) did not report having perceived pressure to do the SAS. One respondent (0.1%) felt pressured by her obstetric professional and seven (0.8%) respondents felt pressure from their partner or social environment to do the SAS. 21.0% (*n*=188) of the women reported that they were advised by their obstetric professional to have the SAS. The main reasons for the respondents to have the SAS were to be reassured about the health of their unborn child (38.7%, *n*=346) and to be confirmed that their child is healthy (37.9%, *n*=339).

## Discussion

This study investigated the level of informed choice of Dutch pregnant women opting for the SAS. An informed choice was made by 54.8% of the respondents. The majority of women had sufficient knowledge about the SAS, did not perceive any pressure to do the SAS and experienced low decisional conflict.

The relatively low informed choice rate of 54.8% was mainly the result of no deliberation. It has been described previously that not all pregnant women realize that having a SAS is an autonomous choice [33]. Literature concerning the level of informed choice for the SAS using the adapted multidimensional measure of informed choice (MMIC), is, however, scarce. A Swedish study that evaluated the effects of an informational film on making an informed choice for the SAS, found an informed choice rate of 81.3% in the intervention group and 76.1% in the control group [34]. Deliberation was, however, not measured in this study and therefore may have resulted in higher rates of informed choice. Other studies focusing on knowledge and expectations of women towards the SAS or aneuploidy screening, report the knowledge of women about the screening test as good [35, 36] or unsatisfactory [37, 38]. Despite a relatively low informed-choice rate in this study, most pregnant women (89.6%) did have sufficient knowledge about the SAS and the majority (92.7%) were classified as value-consistent. Women with low education and women of non-Western descent, were less likely to make an informed choice. Informed choice was associated with less decisional conflict, but decisional conflict was low in both women who made an uninformed and informed choice. Importantly, the large majority did not perceive pressure to do the SAS. Furthermore, women with low education or respondents of non-Western ethnicity were significantly less likely to make an informed choice. This is in line with previous research showing that women with lower levels of education [12, 15, 17, 30, 34, 39, 40] or women from ethnic minority groups less often make an informed choice regarding prenatal testing [17, 30, 39–41]. As informed choice is desirable in these groups, it has to be investigated which specific needs and interventions help to increase informed decision making in women from different cultural groups or with lower levels of education. In our study, more women were multiparous (62.0%) compared to primiparous. The relatively low rate of informed choice might therefore be surprising. Multiparous women might have acquired sufficient knowledge about the SAS from a previous pregnancy, but possibly did not deliberate the decision for the SAS in the current pregnancy anymore.

A higher informed choice rate (76.8%) for women having the non-invasive prenatal test (NIPT) or first-trimester combined test was found by Van der Meij et al. [15]. The difference in level of informed choice between fetal aneuploidy screening and the SAS might be explained by the views of pregnant women on these screening tests. Fetal aneuploidy screening screens for well-defined and known conditions, such as for Down’s, Edwards’ and Patau’s syndromes [15]. It provides parents with the opportunity to make a decision about continuing or terminating the pregnancy, whereas the SAS encompasses more elements than reproductive decision making. It screens for many conditions, including milder and treatable conditions. Furthermore, it adds the element of maternal bonding such as seeing the unborn child and the possibility of knowing the sex. In a study about the perceived value of prenatal ultrasound screening, it has been reported that women valued ultrasound examination to give knowledge about the health of their child and to prepare themselves in case of congenital anomalies [42]. Another possible explanation for the difference in informed choice rate might be the reimbursement for prenatal screening in the Netherlands, whereas the SAS is fully covered, but for the NIPT women need to pay 175 euros [15, 23]. Because of these costs, it could be that a larger proportion of pregnant women deliberate their choice for the NIPT.

Of the women who made an uninformed choice for the SAS in our study, 77.1% was attributed to the absence of deliberation. Deliberation is the process of evaluating the alternatives and weighing up pros and cons [28]. When women or couples do not deliberate their choice for prenatal screening, this has been described as routinization of the decision [21]. It is thought that routinization of prenatal screening might negatively influence prenatal counselling and decision making by offering less or incomplete information to pregnant women, presenting the screening as standard procedure or by counselling women directively. Furthermore, routinization might generate social pressure to test [21]. However, we believe that in our study, the absence of deliberation in women who made an uninformed choice did not negatively impact the decision making for the SAS, because most women had sufficient knowledge about the SAS and experienced low decisional conflict. In addition, although 21.0% of the women in our study reported that they were advised by their obstetric professional to undergo the SAS, most women did not perceive pressure to do the SAS, indicating that the majority was able to make an autonomous decision. Previous studies reported that Dutch women have not felt pressured by society or others to accept or decline prenatal screening [15] and women tend to make their decision about the SAS by themselves or together with their partner [34]. We further found that pregnant women who made an informed choice for the SAS experienced less decisional conflict about their decision making compared to women who made an uninformed choice, which is in accordance with previous studies [11, 12, 14, 17].

### Strengths and limitations

Strengths of this study include the large study sample and the inclusion of pregnant women from all regions in the Netherlands. Another strength is that the participants completed the survey before receiving the SAS. Furthermore, the response rate was high (80.1%) among women who received a questionnaire. A limitation of this study is the small size of women with a non-western background or low level of education and the large proportion of women with a high level of education. Also, our study did not include women who refrained from the SAS. Furthermore, it is important to note that, although the MMIC is often used as a measure of informed choice, scales and cut-offs vary between studies [12, 17, 30, 40, 43]. Therefore, results should be interpreted with caution.

## Conclusion

Our study showed a relatively low rate of informed choice for the SAS, due to absence of deliberation. Therefore, some routinization seem to be present in the Netherlands. However, the majority of women had sufficient knowledge about the SAS, did not perceive pressure to do the SAS and experienced low decisional conflict. 

### Supplementary Information

**Additional file 1.**

## Data Availability

The datasets used and/or analysed during the current study are available from the corresponding author on reasonable request.

## Abbreviations

SAS: Second-trimester anomaly scan
GA: Gestational age
METC LDD: Medical ethical review committee Leiden-Den Haag-Delft
MMIC: Multidimensional measure of informed choice
DCS: Decisional Conflict Scale
SD: Standard Deviation
IQR: Interquartile range
NIPT: Non-invasive prenatal test
