# Supplementary material for: Informed choice and routinization of the second-trimester anomaly scan: a national cohort study in the Netherlands
Source: BMC Pregnancy Childbirth. 2023 Sep 26;23:694. doi: 10.1186/s12884-023-05981-z (PMC10521538; doi:10.1186/s12884-023-05981-z)
Supplement: Supplementary file 1 — Additional file 1. [file 12884_2023_5981_MOESM1_ESM.docx]

**Appendix A: Questionnaire items**

Knowledge measure – developed by the multidisciplinary research team and based on literature^2, 25^

|  |  |
| --- | --- |
| The aim of the SAS is to detect structural anomalies in the unborn child | □ True  □ False  □ I do not know |
| The chance that an unborn child has a congenital anomaly is generally relatively small (less than 5%) | □ True  □ False  □ I do not know |
| Every pregnant woman in the Netherlands is obligated to have a SAS | □ True  □ False  □ I do not know |
| Repeatedly performing a scan is dangerous for the unborn child | □ True  □ False  □ I do not know |
| When finding an anomaly during the SAS, further examination is required to obtain more certainty | □ True  □ False  □ I do not know |
| When finding an anomaly during the SAS, further examination is mandatory | □ True  □ False  □ I do not know |

Attitude measure – based on literature^10, 15^

| In my opinion, having a SAS during my pregnancy, is.. | | | | | | |
| --- | --- | --- | --- | --- | --- | --- |
| Negative  Difficult  Frightening  Not Reassuring | □  □  □  □ | □  □  □  □ | □  □  □  □ | □  □  □  □ | □  □  □  □ | Positive  Easy  Not frightening  Reassuring |

Deliberation scale– based on literature^24^

|  | Strongly disagree | Disagree | Neither agree or disagree | Agree | Strongly agree |
| --- | --- | --- | --- | --- | --- |
| I have tried to mentally visualize the situation | □ | □ | □ | □ | □ |
| I have imagined how I would feel if I did not did not participate in the SAS | □ | □ | □ | □ | □ |
| I have imagined how I would feel if I did participate in the SAS | □ | □ | □ | □ | □ |
| I have tried to think through the consequences of not accepting the SAS | □ | □ | □ | □ | □ |
| I have tried to think through the consequences of accepting the SAS | □ | □ | □ | □ | □ |
| I have made a (mental) list of the pros and cons of the SAS | □ | □ | □ | □ | □ |

Decisional Conflict Scale – validated questionnaire ^27, 28^

|  | Strongly agree | Agree | Neither agree or disagree | Disagree | Strongly disagree |
| --- | --- | --- | --- | --- | --- |
| I know which options are available to me | □ | □ | □ | □ | □ |
| I know the benefits of each option | □ | □ | □ | □ | □ |
| I know the risks and side effects of each option | □ | □ | □ | □ | □ |
| I am clear about which benefits matter most to me | □ | □ | □ | □ | □ |
| I am clear about which risks and side effects matter most | □ | □ | □ | □ | □ |
| I am clear about which is more important to me (the benefits or the risks and side effects) | □ | □ | □ | □ | □ |
| I have enough support form others to make a choice | □ | □ | □ | □ | □ |
| I am choosing without pressure from others | □ | □ | □ | □ | □ |
| I have enough advice to make a choice | □ | □ | □ | □ | □ |
| I am clear about the best choice for me | □ | □ | □ | □ | □ |
| I feel sure about what to choose | □ | □ | □ | □ | □ |
| This decision is easy for me to make | □ | □ | □ | □ | □ |
| I feel I have made an informed choice | □ | □ | □ | □ | □ |
| My decision shows what is important to me | □ | □ | □ | □ | □ |
| I expect to stick with my decision | □ | □ | □ | □ | □ |
| I am satisfied with my decision | □ | □ | □ | □ | □ |

Decision making – developed by the multidisciplinary research team

|  |  |
| --- | --- |
| Have you experienced pressure from your environment to have the SAS done?^†^ | No, I didn't feel any pressure |
|  | Yes, by my partner |
|  | Yes, from my friends/family |
|  | Yes, by my gynecologist/midwife |
|  |  |
| Did your gynecologist/midwife advise you about whether or not to have the SAS? | Yes, to do the SAS |
|  | Yes, not to do the SAS |
|  | No, no advice but he/she was positive about the SAS |
|  | No, no advice but he/she was negative towards the SAS |
|  | No, no advice and he/she didn't show what he/she thought |
|  |  |
| What is the most important reason for you to have a SAS? | To be reassured about my child's health |
|  | To get confirmation that my child is healthy |
|  | To detect physical anomalies |
|  | To see my child one more time |
|  | To see if it's a boy or a girl |
|  | To get a picture of my child |
|  | To get a better bond with my child |
|  | So my partner can see our child |
|  | Because my friends have done it too |
|  | Because my gynecologist/midwife thought it was a good idea |
|  | I do not know |
|  | Other |
|  |  |

^†^It was possible to give multiple answers for this question
